# Pheophorbide *a*: State of the Art

**DOI:** 10.3390/md18050257

**Published:** 2020-05-14

**Authors:** Assunta Saide, Chiara Lauritano, Adrianna Ianora

**Affiliations:** Marine Biotechnology Department, Stazione Zoologica Anton Dohrn, 80121 Naples, Italy; adrianna.ianora@szn.it

**Keywords:** microalgae, chlorophyll, anticancer compound, pheophorbide *a*, cell death

## Abstract

Chlorophyll breakdown products are usually studied for their antioxidant and anti-inflammatory activities. The chlorophyll derivative Pheophorbide *a* (PPB*a*) is a photosensitizer that can induce significant anti-proliferative effects in several human cancer cell lines. Cancer is a leading cause of death worldwide, accounting for about 9.6 million deaths, in 2018 alone. Hence, it is crucial to monitor emergent compounds that show significant anticancer activity and advance them into clinical trials. In this review, we analyze the anticancer activity of PPB*a* with or without photodynamic therapy and also conjugated with or without other chemotherapic drugs, highlighting the capacity of PPB*a* to overcome multidrug resistance. We also report other activities of PPB*a* and different pathways that it can activate, showing its possible applications for the treatment of human pathologies.

## 1. Introduction

Microalgae are photosynthetic microscopic unicellular organisms able to convert solar energy into chemical energy [1]. They can generally be grown with simple cultivation techniques and their biomass can be used to produce human dietary supplements, animal feeds and other beneficial substances for human applications, due to their high levels of proteins, vitamins, pigments and essential amino acids which are not synthesized by the human body [2,3]. 

Marine microalgae have attracted increasing interest due to the possibility of cultivating them in large quantities in an eco-friendly manner, thus overcoming the problem of supply for chemical and bioactivity characterization. This is a property of particular significance, considering the rising need for new bioactive compounds for pharmaceutical applications, due to the increasing incidence of cancer, infectious diseases, viral infections, antibiotics resistance and other human pathologies, [4]. Recent studies showed that microalgae produce metabolites with anticancer [5], anti-inflammatory [6], anti-diabetes [7], antioxidant [8], anti-tuberculosis [9], anti-epilepsy [10], anti-hypertensive [8], anti-atherosclerosis [8] anti-osteoporosis [8] and immunomodulatory activities [11,12]. In addition, various authors showed that different nutrient culturing conditions could influence microalgae bioactivities [6,13], triggering the activation of specific metabolic pathways [14,15,16,17]. These studies showed that the bioactivity of microalgae is not correlated with a specific microalgae class or a specific culturing condition, but depends on the microalgae clone, the algal growth phase, the culturing condition and chemical extraction protocol used, and should be carefully considered case by case. There are already several microalgae products on the market, but only for cosmeceutical and nutraceutical applications (such as moisturizing and anti-aging creams or microalgae-based food supplements) [18]. Microalgae have also been shown to provide promising opportunities in the field of functional foods, due to their production of valuable bioactive ingredients, such as carotenoids, with already known health benefits [18]. Furthermore, they have been recognized as the most prolific photoautotrophic producers of pigments, such as chlorophyll, one of the most important natural pigments extracted from microalgae biomass, used as a natural edible dye agent with antioxidant and anti-mutagenic properties [1]. 

Many studies have been carried out to optimize chlorophyll extraction and fractionation from microalgae [1]. The extraction process begins with the dewatering and desalting of the microalgae cultures (biomass concentration = 0.1–1% w/v). Chlorophyll is then extracted from the dried biomass by organic solvent extraction or supercritical fluid extraction. These processes are followed by a fractionation step to separate chlorophyll pigments and derivatives. 

There are four types of chlorophylls found in marine algae. The first is chlorophyll *a* which absorbs most energy from the violet blue and orange-red light wavelengths [19]. Chlorophyll *a* is the crucial molecule for photosynthesis through the passing of its energized electrons on to molecules, which will synthesize the sugars. Chlorophyll *b* helps in photosynthesis by absorbing light energy. It is more soluble than chlorophyll *a* in polar solvents, because of its carbonyl group. Chlorophyll *c* is an accessory pigment with a similar function to chlorophyll *b* to expand the number of wavelengths of light that can be absorbed for photosynthesis. Chlorophyll containing light–harvesting complexes (LHCs) in chloroplasts of plants and algal cells usually include chlorophyll *b* or *c* in addition to chlorophyll *a.* Chlorophyll *d* was first described in 1943 (Manning WM, Strain HH (November 1943)) [20] in cyanobacteria that absorb far-red light, at 710 nm wavelength, due to their adaptation to deep water, where there is not much visible light for photosynthesis [21]. 

Humans convert chlorophylls into phaeophytin, pyrophaeophytin or pheophorbide during the digestion of vegetables. Chlorophyll and its derivative products are known for their health benefits, and not only function as colorants, but also possess antioxidant and potential therapeutic properties [22,23]. In this review, we discuss the activity of pheophorbide *a (*PPB*a),* one of the most extensively studied chlorophyll products because it is a good photosensitizer, compared to others such as the widely used Photofrin in clinical treatments, a mixture of hematoporphyrines, but which has a weak absorption above 600 nm [24]. PPB*a* is characterized by a strong absorption between 650–700 nm, in the tissue-penetrating wavelength range; Rapozzi et al. found that PPB*a* irradiated at 14 J/cm^2^ induces a potent photodynamic effect in different cancer cells with IC_50_ values between 70 and 200 nM [25]. In this review, we describe PPB*a* bioactivity and possible applications for the treatment of cancer and other human pathologies.

## 2. Pheophorbide *a* and its Synthesis

**Figure 1:**
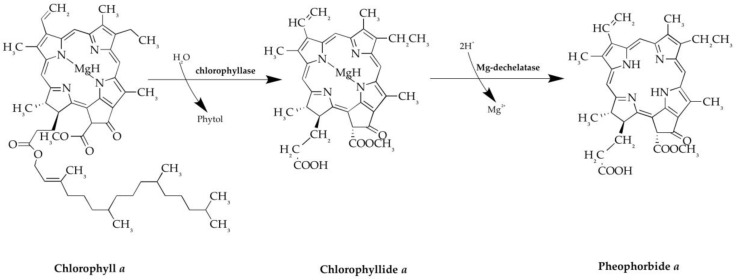
Synthesis of Pheophorbide *a*, from Chlorophyll *a.* Chlorophyllase and Mg-dechelatase enzymes catalyze the reaction.

During the period 1996–2014, many studies have focused on this compound because PPB*a* complies with most criteria that a good photosensitizer should satisfy. PPB*a* is the dephytylation and demetallation product of chlorophyll *a*, which is formed in algae and higher plants. This catabolic transformation is mediated by chlorophyllase and Mg-dechelatase. PPB*a* is composed by a tetrapyrrolic microcycle bearing four methyls, one ethyl, one vinyl, one methoxycarbonyl and one propionyl as substituents (Figure 1). The UV-vis spectrum of PPB*a* is typical of chlorophyll *a* type compounds, with a Soret band at 390 nm and Q-bands between 500 and 700 nm. The 670-nm Q-band is of relatively high intensity and makes PPB*a* an attractive molecule for Photodynamic therapy (PDT) [26].

## 3. Photodynamic Therapy

In 1996 Schuitmaker et al. [27] reported the important action of photodynamic therapy (PDT) that was, already in the early 1980s, described as a “promising new modality in the treatment of cancer". In particular, they highlighted the effect of two therapeutic agents combined, a photosensitizing drug and light, which were relatively innocuous by themselves but, when mixed, selectively caused tumor destruction. The therapeutic properties of light have been known for thousands of years, but it was only in the last century that PDT was developed. PDT involves two individually non-toxic components that are combined to induce cellular and tissue effects in an oxygen-dependent manner. The first component of PDT is a photosensitizer that is a photosensitive molecule that localizes a target cell and/or a tissue. The second component involves the administration of light of specific wavelength that activates the sensitizer. The photosensitizer transfers energy from light to molecular oxygen, to generate reactive oxygen species (ROS) [28]. It is known that there are three major mechanisms by which PDT mediates tumor destruction [29]: (1) ROS that are generated by PDT can directly kill tumor cells; (2) PDT also damages the tumor-associated vasculature, leading to tumor restriction; (3) Finally, PDT can activate an immune response against tumor cells. These three mechanisms can also influence and activate each other. PPB*a* was found to be active in PDT, as explained in detail in the following paragraph.

## 4. Bioactivities of Pheophorbide *a* (PPB*a*)

### 4.1. Anti-cancer Activity

PPB*a* has been shown to have antiproliferative activity versus various cell lines (e.g., Jurkat-human T lymphocyte from acute T cell leukemia; MES-SA-human uterus from uterine sarcoma; HepG2-human liver from hepatocellular carcinoma; Hep3B-human liver from hepatocellular carcinoma; MDA-MB-231-human mammary gland/breast adenocarcinoma; MCF-7-human mammary gland/breast adenocarcinoma; YD-10B- human oral squamous from carcinoma; HT29-human colon from colorectal adenocarcinoma; HCT116-human colon from colorectal carcinoma; U87MG-human brain from likely glioblastoma), with or without PDT.

In 1996, Nakamura et al. [30] demonstrated, by using an in vitro assay*,* that vegetables were inhibitors of Burkitt lymphoma tumor-promoter induced Epstein Barr virus (EBV) activation [31]. In their study, they focused on the water mimosa *Neptunia oleracea* (Leguminosae), which is used as a sour vegetable in Thailand, and identified different compounds, including PPB*a* and pheophorbide *b* (PPB*b*). They also showed that these compounds had a marked inhibitory activity toward EBV activation at a concentration of 5 μM. The IC_50_ inhibitions were 3.3 and 4.5 μM respectively and the inhibitory activities were from 5 to 10 times higher than those of other compounds (pheophorbide *a* ethyl ester, pheophorbide *b* ethyl ester, pheophorbide *a* methyl ester, 10-hydroxy-pheophorbide *a*) extracted from the same plant. In particular, PPB*a* and 10-hydroxy-pheophorbide *a* are known as dietary photosensitizers. However, in this case, after the irradiation of Raji cells (Burkitt lymphoma cell line that is latently infected with EBV) [32] with 10.000 lux for 5 min these compounds did not show any significant difference in inhibitory activity, with or without irradiation. 

Tang et al. [33] revealed the antiproliferative effect of PPB*a/*PDT on human hepatocellular carcinoma Hep3B cells (ATCC®HB-8064^TM^). The activity was dose-dependent with an IC_50_ value of 1.5 μM and only with PDT. The same authors in 2009 demonstrated that using PPB*a* in PDT significantly inhibited the growth of human uterine sarcoma cell line MES-SA (ATCC®CRL-1976^TM^), with an IC_50_ value of 0.5 μM at 24 h [34]. No cytotoxicity was observed when no light illumination was applied. In both papers, the results demonstrated that PPB*a/*PDT significantly inhibited the growth of the cancer cells by the induction of programmed cell death via the mitochondrial dependent apoptotic pathway, suggesting that PPB*a/*PDT can potentially be an effective protocol for uterine carcinosarcoma and hepatocellular carcinoma treatment [33,34].

Successively, Tang et al. [35] with their findings provided the first evidence that PDT could inhibit multidrug resistance (MDR, i.e., resistance frequently observed after prolonged cancer treatment with conventional anti-tumor drugs) activity by down-regulating the expression of P-glycoprotein via c-Jun N-terminal kinase (JNK) pathway activation, using PPB*a* as the photosensitizer. Their work proved that PPB*a/*PDT inhibited the growth of MDR hepatoma cells (R-HepG2) when cell cultures were treated with Doxorubicin from 0.1 to 100 μM that induced mitochondrial-mediated apoptosis. As with Raji, Jurkat, MES-SA and Hep3B cancer human cells treatment with PPB*a/*PDT exhibited a potent anti-tumor effect on MDA-MB-231 human breast adenocarcinoma, inhibiting cell growth with an IC_50_ value of 0.5 μM after 24 h of treatment [36]. 

Hoi et al. [37] demonstrated that PPB*a/*PDT leads to apoptosis in MCF-7 (human breast tumor cell line ATCC®HTB-22^TM^). MCF-7 is a human breast tumor cell line that differs from MDA-MB-231 due to the presence of estrogen receptors. The cytotoxic activity of PPB*a*/PDT (IC_50_ = 0.5 μM) induced cell death via both caspase-dependent and -independent apoptotic pathways [37]. The two last cited studies [36,37] showed that PPB*a* does not compete with 17β-estradiol for the estrogen receptor binding, because the effect is the same in both cellular lines, with and without estrogen receptors. Hoi et al. [37] also tested the effects of PPB*a/*PDT in vivo using female BALB/c nude mice, showing that an intravenous injection of 2.5 mg/kg of PPB*a/*PDT in mice could exhibit a dramatic and significant (*p* < 0.001) regression of MCF-7 cells in the xenograft model.

Ahn et al. [38,39] demonstrated the effects of PPB*a*/PDT in vitro and in vivo*,* on human oral cancer. In their first study in 2013, Ahn et al. worked on a human oral squamous cell line (YD-10B, purchased from Korea Cell Line Bank), demonstrating that PPB*a*/PDT inhibited cell proliferation after 24 h of treatment in a dose-dependent manner up to 2 μM. They demonstrated that cell death was due to an apoptotic process through a mitochondria-dependent intrinsic pathway. Moreover, they showed that PPB*a*/PDT dramatically inhibited the phosphorylation of extracellular signal-regulated kinases (ERK) and that this action caused apoptotic cell death by cytotoxic ROS generation [38]. In 2017, Ahn et al. investigated the therapeutic effect of PDT using intratumoral administration of the PPB*a* in an in vivo murine oral squamous cell carcinoma (OSCC) animal model (male immunocompetent C3H mice from Samtaco, Sungnam, South Korea). PPB*a* was intratumorally administered to mice inoculated with AT-84 cells (murine OSCC cell lines). One week later, the animals were administered PPB*a* intratumorally at a dosage of 10 mg/kg. After 2 h, PDT was performed using a laser diode at a light dose of 100 j/cm^2^. They observed that the intratumoral PPB*a*/PDT significantly decreased tumor volume compared to the control group and inhibited tumor growth up to 60%, relative to the control group. In this study the levels of Proliferating Cell Nuclear Antigen (PCNA) and Bcl-2 expression were markedly decreased, whereas Caspase 3 and PARP cleavage activation was increased [39]. These findings suggest that intratumoral PPB*a*/PDT could be a potent clinical therapeutic strategy for OSCC. 

Cho et al., in 2014, showed that PPB*a* (0–20 μg/mL) significantly reduced human glioblastoma cells U87MG (ATCC®HTB14^TM^) growth, in a dose-dependent manner, in the absence of direct photo-irradiation. There was a strong antiproliferative effect (IC_50_ = 2.8 μg/mL), but no cytotoxic effect, on normal endothelial HUVEC cells (HUVEC cells were obtained from human umbilical cord veins). The glioblastoma growth inhibitory activity of PPB*a* was associated with cell cycle arrest in the G0/G1 phase and apoptosis, with the degradation of genomic DNA. Hence, the results suggested that PPB*a* isolated from the marine alga *Grateloupia elliptica* could be a good source for glioblastoma-specific therapy with no clinical side effects [40].

The anticancer activities of PPB*a* are summarized in Table 1.

### 4.2. Pheophorbide a Conjugated with Anticancer Drugs

In 2011, You [41] found that PPB*a* conjugated with anticancer drugs, such as Doxorubicin (DOX) and Paclitaxel (PTX), could be utilized for selective cancer therapy as well as for the fluorescence detection of cancer. The study reported that the fluorescence of PPB*a* and DOX conjugated by excitation at 420 or 440 nm was greatly diminished, possibly by the energy transfer mechanism between the two fluorescent groups. However, upon treatment in cancer cells, the conjugate was cleaved to restore both fluorescent groups of PPB*a* and DOX after 48 h of incubation. They also reported the effects of PPB*a* conjugated with anticancer drugs, either by direct coupling or via linkers against different cell lines: MCF 7 (breast adenocarcinoma), KB (mouth carcinoma), HeLa (cervical cancer), U-87MG (glioblastoma), A549 (lung adenocarcinoma), AT-84 (oral cancer), and YD-10B (oral cancer). They showed that each linker could exert antiproliferative activities in specific cancer cells. Hence, to be active, the photosensitizer and anticancer drugs need specific linkers.

The cells were incubated for three days with synthesized compounds and the viability was measured using the SRB assay (Sulforhodamine B assay in cell culture to investigate cell proliferation). Regarding direct coupling, You [41] found that 10 μM of PPB*a/*DOX moderately inhibited the growth of cancer cells, including MCF-7, HeLa, U87MG, and AT-84 cells, but it had lower activity compared to DOX alone. On the other hand, PPB*a-*PTX showed potent inhibitory activity of the viability of MCF7, HeLa, KB and YD 10B cells at 10 μM.

Regarding the use of linkers, hydroxycinnamoyl (PPB*a*/DOX) moiety and aminobenzyloxycarbonyl (PPB*a*/DOX) moiety, You [41] showed that both PPB*a*/DOX conjugated with linkers did not exert significant activities (Table 2). 

In 2017, Ruiz-González et al. [42] demonstrated with three different combined treatments of PPB*a*, red light and doxorubicin (DOX) that the timing with which the drugs and light were administered plays a crucial role in the final outcome of the treatments, ranging from antagonistic to synergic for the same concentrations. The three protocols used to identify the most active conditions were: (1) cells were incubated with PPB*a* for 4 h, washed to remove any unbound PPB*a*, irradiated for 15 min and then further incubated with DOX for 24 h. (2) Cells were first treated with DOX for 24 h, washed, incubated with PPB*a* for 4 h, washed again and finally irradiated for 15 min. (3) Cells were incubated with DOX for 20 h, washed, co-incubated with PPB*a* and DOX for 4 h, washed again and finally irradiated for 15 min. For all three protocols, they used 2 μM of PPB*a* and 0.4 μM of DOX. The results showed that the third protocol induced 25% greater cell survival than the other two (40%), compared to DOX individual treatments (48%) or PPB*a/*PDT (46%) used as control [37]. 

The combination of chemotherapeutic drugs with photosensitizing agents is an increasingly growing area of study in vitro and in vivo, and even in clinical trials aiming to overcome multidrug resistance.

### 4.3. Other Bioactivities of PPBa

Different bioactivities (i.e., antiviral, anti-inflammatory, antioxidant, immunostimulatory, anti-parasite activities) have been associated to PPB*a* (Table 3).

#### 4.3.1. Antiviral Activity

In 1998, Ohta et al. [43] studied 100 microalgae comprising 77 marine and 23 freshwater algae for new antiviral compounds. Algae were grouped into six classes, as follows: 55 *Cyanophyceae*, 37 *Chlorophyceae*, 4 *Bacillariophyceae, Criptophyceae,* 2 *Chrysophyceae,* and *Rhodophyceae*. These were tested on Vero cells (ATCC®-CCL81^TM^) monolayers obtained from the Japanese Cancer Research Resources Bank. The antiviral activity against adeno-virus (Type VI strain, Ad75), herpes simplex virus (HSV-1 and 2, Type I HF strains), Japanese Encephalitis (JAGAO 01 strain) and Polio virus, obtained from Osaka prefectural Institute of Public health of Japan, was determined by microscope observation of the inhibition of the appearance of the cytopathic effect in Vero cells (i.e. structural changes in host cells caused by viral invasion). They observed that the cytopathic effect of herpes simplex virus, Type 1 and 2 was inhibited by four methanol extracts of *Dunaliella bioculata* C-523, *Dunaliella primolecta* C-525, *Lyngbya sp*. M-9 and *Lyngbya aeruginro-coerulea* M-12. The green alga, *Dunaliella primolecta*, had the highest anti-Herpes Simplex virus Type 1 and 2 activities, since 10 μg/mL^−1^ of extract from this alga completely inhibited the cytophatic effect. Thus, the activity was specific against HSV. 

By using various chromatographic techniques, they purified three green compounds having anti-HSV activity from the algal biomass of *D. primolecta;* these purified compounds completely inhibited the cytophatic effect when tested at *5* μg/mL. From the analysis of NMR and MS, the chemical structures of the active substances were identified as pheophorbide-like compounds [43]. Similarly, Ratnoglik et al. [44] demonstrated that PPB*a* extracted from the terrestrial plant *Morinda citrifolia,* and its related compound pyropheophorbide *a*, could be good candidates for developing antivirals against Hepatitis C virus (HCV), because they showed potent anti-HCV activities, with an IC_50_ of 0.3 μg/mL for PPB*a* and 0.2 μg/mL for pyropheophorbide *a*.

#### 4.3.2. Anti-inflammatory Activity 

Inflammation is involved in several human pathologies; it is a protective response that involves immune cells, blood vessels, and different molecular mediators (e.g., Tumor Necrosis Factor α, Interleukin 1, nitric oxide, and prostaglandins). In recent years, many studies have investigated possible microalgae anti-inflammatory properties [6,45,46]. A recent study by Lauritano et al. [47] investigated the capacity of extracts of the diatom *Cylindrotheca closterium* to inhibit the release of one of the main effectors of inflammation, Tumor Necrosis Factor α, in Lipopolysaccharide-stimulated human THP-1 monocytic leukemia cells (ATCC®TIB-202^TM^). They tested five fractions and identified Fraction C as the most active, able to inhibit 60% of Tumor Necrosis Factor α release at 100 μg/mL and 40% of Tumor Necrosis Factor α release at 50 μg/mL concentration. Fraction D also showed activity (40% at 100 μg/mL and 30% Tumor Necrosis Factor α release at 50 μg/mL). After dereplication and further characterization of the two active fractions by UHPLC-HR-MS/MS in order to identify the potential bioactive compounds responsible for the observed anti-inflammatory activity, they found lysophosphatidylcholines in Fraction C (1-palmitoyl-sn- glycerol-3-phosphocoline was the most abundant) and PPB*a* in Fraction D. 

#### 4.3.3. Antioxidant Activity

Many studies have demonstrated the antioxidant activity of derivatives of marine organisms [48,49]. Sakata et al. [50], in 1990, extracted, for the first time, a new PPB*a* related compound named as Chlorophyllone-A, that was isolated as an antioxidant compound from the extract of the short-necked clam, *Ruditapes philippinarum* (probably a diet derived compound), suggesting a new degradation pathway of chlorophylls. The extract of the clam *Ruditapes philippinarum* showed very low peroxidant values analyzed by using the antioxidant ferric thiocyanate method. 

In 2005, Lanfer-Marquez [51] evaluated the protective action of six natural purified chlorophyll derivatives (chlorophyll *a* and *b*, pheophytin *a* and *b*, pheophorbide *a* and *b*) extracted from 5 g dehydrated spinach, but also the synthetic Cu-chlorophyllin against lipid oxidation employing two methods: the bleaching of β-carotene in a water/linoleic acid emulsion and the scavenging of the stable radical 2,2-di-phenyl-1-picrylhydrazyl (DPPH). The results obtained by the β-carotene bleaching method showed that all chlorophyll derivatives presented a dose-dependent response; in particular PPB*a* presented a weak antioxidant activity, starting slowly at 12.5 ppm and reaching its highest activity at 150 ppm, which was lower than Pheophorbide *b*. 

In 2011, Cho et al. [52] showed the antioxidant property of the green alga *Enteromorpha prolifera* that was evaluated after ethanol extraction and sub-fractionation, using solvent partitioning and chromatography. The ethanol extract and its solvent subfractions, partitioned by *n*-hexane (HX), chloroform (CF) and ethylacetate (EA), were measured for antioxidant activities*;* the CF fraction showed the most potent 2,2-diphenyl-1-picrylhydrazyl (DPPH) and hydroxyl (OH) radical scavenging capacities that were comparable to the positive control, butylated hydroxyanisole and α-tocopherol, at concentrations from 0.25 to 1.0 mg/mL. Further elucidation of the CF subfractions using various spectroscopic techniques revealed that the major compound contributing to the antioxidant activity was PPB*a*, rather than phenolic compounds.

#### 4.3.4. Immunostimulatory Activity

In 2011, Bui-Xuan et al. [53] revealed for the first time that PPB*a* without PDT possessed immunostimulatory effects on the murine macrophage cell line RAW 264.7. Low dosages of PPB*a* (0–1 μM) significantly stimulated the growth of RAW 264.7 cells, with maximum effects at 1 μM after 24, 48, and 72 h of treatment (*p* < 0.05 for all the analyses). In addition, intracellular mitogen activated protein kinases (MAPK), including extracellular signal-regulated kinase (ERK) and p38 MAPK, were activated by PPB*a* treatment in a dose dependent manner. Furthermore, PPB*a* significantly induced the release of interleukine-6 and tumor necrosis factor-α, and enhanced the phagocytic activity of RAW 264.7 cells (*p* < 0.05 for all the analyses). The stimulation of the growth of macrophages can reinforce the immune defense of the host. The production of pro-inflammatory cytokines and enhancement of phagocytic capacity could be beneficial effects for tumor clearance upon chemotherapy or photodynamic therapy [36]. 

#### 4.3.5. Anti-parasite Activity

Many studies have explored PDT as an alternative for the treatment of cutaneous leishmaniasis. There are different efficient photo-sensitizers for the treatment of this disease, such as methylene blue [54] and carbaporphyrin ketals [55]. Miranda et al. [56] in 2017 elucidated the mechanism of action of PPB*a* in promastigotes and intracellular amastigotes (two developmental stage during the growth phases) of *Leishmaniasis amazonensis*, the parasite responsible for the disease leishmaniasis. Authors showed that PPB*a*/PDT inhibited the proliferation of promastigotes and amastigotes. Interestingly, PPB*a* activity was stronger against intracellular amastigotes, which is the clinically most relevant form of leishmaniasis (intracellular amastigotes: IC_50_ = 1.01 μM; promastigotes: IC_50_ = 17.9 μM). The presence of light was fundamental for the activity of the compound. PPB*a* caused strong impairment in redox system homeostasis and alterations in mitochondrial membrane potential [ΔΨ_m_]. These alterations caused cell death in both promastigotes and intracellular amastigotes forms.

## 5. Conclusions

Marine microalgae are considered a potentially new and valuable source of biologically active molecules for applications in the food industry, as well as in the pharmaceutical, nutraceutical, and cosmetic sectors [6]. Chlorophyll-derived molecules are valuable bioactive compounds that can be extracted from microalgal biomass. The exposure of chlorophyll-derived molecules to weak acids, oxygen or light, accelerate their oxidation and result in the formation of numerous other degradation products which can have antioxidant, anticancer, anti-inflammatory, anti-obesity, neuroprotective and antiangiogenic properties [57]. 

In particular, PPB*a*, one of the products derived from chlorophyll degradation, has attracted widespread attention in recent years as a non-invasive and highly selective approach for cancer treatment. PPB*a* has been shown to be a good photosensitizer and has been usually used in combination with photodynamic therapy. In addition, several trials have been performed combining PPB*a* to chemotherapic drugs (e.g., Doxorubicin) showing interesting activities [58]. Several antibiotics of the anthracycline group, such as Doxorubicin, acting as DNA intercalators and inhibitors of Topoisomerase II activity, are in use for the treatment of a wide range of cancers [59]. The pathways activated in cancer cell lines after treatment with PPB*a* are generally the activation of the extrinsic and intrinsic apoptotic pathway, the c-Jun-N-terminal kinase pathway and the inactivation of the extracellular signal-regulated kinase pathway.

In summary, the review reports that the combination of a chemotherapeutic drug with a photosensitizing agent could be a possible way to overcome multidrug resistance (MDR). MDR continues to pose a significant challenge in the management of cancer, and it is therefore crucial to understand the molecular basis underlying MDR mechanisms [60]. Tang et al. used PPB*a* together with PDT to overcome MDR in Resistant-HepG2. They showed that PPB*a*/PDT induced c-Jun-N-terminal kinases pathway activation with inhibitory effects on MDR by the down-regulation of P-glycoprotein in R-HepG2. In addition, they obtained significant reduction of tumor size in PPB*a*/PDT treated R-Hep-G2-bearing nude mice, with no significant damages in the liver or heart [30]. 

This review also reports other important bioactivities shown for PPB*a*, such as antiviral [43], anti-inflammatory [6,45,46,47], antioxidant [48,49,50,51], immunostimulatory [53] and anti-parasite activities [56]. These data show how this compound can have a plethora of applications for different human pathologies. The possibility to use microalgal fractions rich in PPB*a* can be a very interesting strategy for functional food production and nutraceutical applications as well. 

## Figures and Tables

**Table 1:** Anticancer activity of Pheophorbide *a* against different cancer cell lines, with/without photodynamic therapy.

Source of Pheophorbide *a*	Investigated Activity	Target Cells	Pathway Involved	Active Concentration	References
Extract from *Neptunia Oleracea*	The inhibition of Burkitt Lymphoma tumor-promoter induced EBV	Burkitt Lymphoma (Raji Cells)		IC_50_ 3.3 µM; With or without PDT.	[30]
Extract from *Scutellaria Barbata*	Antiproliferative-activity	Hepatocellular carcinoma (Hep3B)	Extrinsic and intrinsic apoptotic pathway	IC_50_ 1.5 µM at 48 h; With PDT.	[33]
Purchase Frontier Scientific	Antiproliferative activity	Uterine sarcoma (MES-SA)	Intrinsic apoptotic pathway	IC_50_ 0.5 µM at 24 h; With PDT.	[34]
Purchase Frontier Scientific	Multidrug Resistance	Resistant Human Hepatoma cell (R-HepG2)	JNK pathway	IC_50_ 0.6 µM at 24 h; With PDT.	[35]
Purchase Frontier Scientific	Antiproliferative activity	Human breast adenocarcinoma (MDA-MB-231)	JNK pathway	IC_50_ 0.5 µM at 24 h; With PDT	[36]
Purchase Frontier Scientific	Antiproliferative activity	Human Breast tumor (MCF-7)	Extrinsic and intrinsic apoptotic pathway	IC_50_ 0.5 µM at 24 h; With PDT	[37]
Extract from *Scutellaria Barbata*	Antiproliferative activity	Human oral squamous cell carcinoma (YD10B)	Inactivating ERK pathway	IC_50_ 0.5 µM at 24 h; With PDT	[38]
Synthetized from chlorophyll-a	Anticancer activity	Murine oral squamous cell carcinoma (AT-84) and CH3 mice	Apoptotic pathway	IC_50_ 0.25 µM at 24 h; With PDT	[39]
Extract from *Grateloupia elliptica*	Glioblastoma specific-antiproliferative	Glioblastoma cells (U87MG)	Apoptotic pathway	IC_50_ 2.8 µg/mL at 24 h; Without PDT	[40]

**Table 2:** Cell viability percentage by SRB assay. Activity of Pheophorbide *a* (PPB*a*)*,* conjugated with drugs by direct coupling or by linkers, tested on different cell lines MCF 7 (breast adenocarcinoma), KB (mouth carcinoma), HeLa (cervical cancer), U-87MG (glioblastoma), A549 (lung adenocarcinoma), AT-84 (oral cancer), and YD-10B (oral cancer) with 10 μM of PPB*a* for 72 h. (**1**) PPB*a,* (**2**) Doxorubicin-DOX, (**3**) PPB*a/*DOX directly conjugated, (**4**) PPB*a/*PTX directly conjugated, (**5**) PPB*a/*DOX conjugated with hydroxylcinnamoyl moiety, (**6**) PPB*a/*DOX conjugated with aminobenzyloxycarbonyl moiety [41].

Compounds	MCF7	KB	HeLa	U-87MG	A549	AT84	YD10B
**1**	±65%	±98%	±93%	±70%	±100%	±80%	±100%
**2**	±8%	±2%	±5%	±10%	±90%	±10%	±90%
**3**	±55%	±80%	±60%	±30%	±98%	±40%	±70%
**4**	±20%	±1%	±2%	-	±40%	±42%	±5%
**5**	±58%	±92%	±60%	±58%	±93%	±60%	±83%
**6**	±60%	±90%	±70%	±40%	±100%	±50%	±80%

**Table 3:** Antiviral, anti-inflammatory, anti-oxidant, immunostimulatory and anti-parasitic bioactivities of Pheophorbide *a.*

Source of Pheophorbide *a*	Investigated Activity	Pathway Involved and Experimental Model	Active Concentration	References
Compound present in an activity fraction of *Dunaliella primolecta*	Antiviral	Vero cells infected with herpes simplex Type 1 and 2	10 μg mL^−1^	[43]
Compound present in an activity fraction of *Morinda citrifolia*	Antiviral	HCV cell culture system	Pheophorbide *a*: IC_50_ = 0.3 μg/mL Pyropheophorbide *a*: IC_50_ = 0.2 μg/mL	[44]
Compound present in an activity fraction of *Cylindrotheca closterium*	Anti-inflammatory	THP-1 monocytic leukemia cells	Fraction C: 60% of TNFα release at 100 μg/mL and 40% of TNFα release at 50 μg/mL concentration. Fraction D: 40% at 100 μg/mL, 30% at 50 μg/mL	[47]
Extract from Spinach	Anti-oxidant		150 ppm inhibition of oxidation between 70–80%	[51]
Purchase from Frontier Scientific	Immunostimulatory	MAPK pathway in RAW 264.7 cells	0–5 μM, al *p* > 0.05 From 24 to 72 h	[53]
Purchase from Sigma-Aldrich	Anti-parasite	Caspase3/7 Amastigotes and Promastigotes of Leishmaniasis and J774-A1 culture macrophages	Intracellular amastigotes: IC_50_ = 1.01 μM Promastigotes: IC_50_ = 17.9 μM	[56]

## References

[1] Hosikian A., Lim S., Halim R., Danquah M.K. (2010). Chlorophyll extraction from microalgae: A review on the process engineering aspects. Int. J. Chem. Eng..

[2] Nwe Oo Y.Y., Su M.C., Kyaw K.T. (2017). Extraction and determination of chlorophyll content from microalgae. Int. J. Adv. Res. Publ..

[3] Carotenuto Y., Esposito F., Pisano F., Lauritano C., Perna M., Miralto A., Ianora A. (2012). Multi-generation cultivation of the copepod *Calanus helgolandicus* in a re-circulating system. J. Exp. Mar. Biol. Ecol..

[4] Romano G., Costantini M., Sansone C., Lauritano C., Ruocco N., Ianora A. (2017). Marine microorganisms as a promising and sustainable source of bioactive molecules. Mar. Environ. Res..

[5] Martinez Andrade K.A., Lauritano C., Romano G., Ianora A. (2018). Marine microalgae with anti-cancer properties. Mar. Drugs.

[6] Lauritano C., Andersen J.H., Hansen E., Albrigtsen M., Escalera L., Esposito F., Helland K., Hanssen K.Ø., Romano G., Ianora A. (2016). Bioactivity screening of microalgae for antioxidant, anti-inflammatory, anticancer, anti-diabetes, and antibacterial activities. Front. Mar. Sci..

[7] Lauritano C., Ianora A. (2016). Marine organisms with anti-diabetes properties. Mar. Drugs.

[8] Giordano D., Costantini M., Coppola D., Lauritano C., Nunez Pons L., Ruocco N., di Prisco G., Ianora A., Verde C. (2018). Biotechnological applications of bioactive peptides from marine sources. Adv. Microb. Physiol..

[9] Lauritano C., Martìn J., de la Cruz M., Reyes F., Romano G., Ianora A. (2018). First identication of marine diatoms with anti-tuberculosis activity. Sci. Rep..

[10] Brillatz T., Lauritano C., Jacmin M., Khamma S., Marcourt L., Righi D., Romano G., Esposito F., Ianora A., Queiroz E.F. (2018). Zebrafish-based identification of the antiseizure nucleoside inosine from the marine diatom *Skeletonema marinoi*. PLoS ONE.

[11] Riccio G., Lauritano C. (2019). Microalgae with immunomodulatory activities. Mar. Drugs.

[12] Riccio G., De Luca D., Lauritano C. (2020). Monogalactosyldiacylglycerol and sulfolipid synthesis in microalgae. Mar. Drugs.

[13] Ingebrigtsen R.A., Hansen E., Andersen J.H., Eilertsen H.C. (2016). Light and temperature effects on bioactivity in diatoms. J. Appl. Phycol..

[14] Lauritano C., De Luca P., Ferrarini A., Avanzato C., Minio A., Esposito F., Ianora A. (2017). De novo transcriptome of the cosmopolitan dinoflagellate *Amphidinium carterae* to identify enzymes with biotechnological potential. Sci. Rep..

[15] Lauritano C., De Luca P., Amoroso M., Benfatto S., Maestri S., Racioppi C., Esposito F., Ianora A. (2019). New molecular insights on the response of green algae to nitrogen starvation. Sci. Rep..

[16] Vingiani G.M., De Luca P., Ianora A., Dobson A.D.W., Lauritano C. (2019). Microalgal enzymes with biotechnological applications. Mar. Drugs.

[17] Elagoz A.M., Ambrosino L., Lauritano C. (2020). De novo transcriptome of the diatom *Cylindrotheca closterium* identifies genes involved in the metabolism of anti-inflammatory compounds. Sci. Rep..

[18] Galasso C., Corinaldesi C., Sansone C. (2017). Carotenoids from marine organisms: Biological functions and industrial applications. Antioxidants (Basel).

[19] Holdt S.L., Kraan S. (2011). Bioactive compounds in seaweed: Functional food applications and legislation. J. Appl. Phycol..

[20] Manning W.M., Strain H.H. (1943). Chlorophyll d, a green pigment of red algae. J. Biol. Chem..

[21] Larkum A.W., Kuhl M. (2005). Chlorophyll d: The puzzle resolved. Trends Plant Sci..

[22] Christaki E., Bonos E., Florou-Paneri P. (2015). Innovative microalgae pigments as functional ingredients in nutrition. Biotechnol. Adv..

[23] Maadane A., Merghoub N., Ainane T., El Arroussi H., Benhima R., Amzazi S., Bakri Y., Wahby I. (2015). Antioxidant activity of some Moroccan marine microalgae: Pufa profiles, carotenoids and phenolic content. J. Biotechnol..

[24] Rapozzi V., Zacchigna M., Biffi S., Garrovo C., Cateni F., Stebel M., Zorzet S., Bonora G.M., Drioli S., Xodo L.E. (2010). Conjugated PDT drug: Photosensitizing activity and tissue distribution of PEGylated pheophorbide a. Cancer Biol. Ther..

[25] Rapozzi V., Miculan M., Xodo L.E. (2009). Evidence that photoactivated pheophorbide a causes in human cancer cells a photodynamic effect involving lipid peroxidation. Cancer Biol. Ther..

[26] Xodo L.E., Rapozzi V., Zacchigna M., Drioli S., Zorzet S. (2012). The chlorophyll catabolite pheophorbide a as a photosensitizer for the photodynamic therapy. Curr. Med. Chem..

[27] Schuitmaker J.J., Baas P., Leengoed H.L.L.M.v., Meulen F.W.v.d., Star W.M., Zandwijk N.v. (1996). Photodynamic therapy: A promising new modality for the treatment of cancer. J. Photo Chem. Photobiol. B Biol..

[28] Dolmans D.E.J.G.J., Fukumura D., Jain R.K. (2003). Photodynamic therapy for cancer. Nat. Rev. Cancer.

[29] Dougherty T.J., Gomer C.J., Henderson B.W., Jori G., Kessek D., Korbelik M., Moan J., Peng Q. (1998). Photodynamic therapy. J. Natl. Cancer Inst..

[30] Nakamura Y., Murakami A., Koshimizu K., Ohigashi H. (1996). Identification of pheophorbide a and its related compounds as possible anti-tumor promoters in the leaves of *Neptunia oleracea*. Biosci. Biotechnol. Biochem..

[31] Thompson M.P., Kurzrock R. (2004). Epstein-barr virus and cancer. Clin. Cancer Res..

[32] Jankelevich S., Kolman J.L., Bodnar J.W., Miller G. (1992). A nuclear matrix attachment region organizes the epstein-barr viral plasmid in Raji cells into a single DNA domain. EMBO J..

[33] Tang P.M., Chan J.Y., Au S.W., Kong S.K., Tsui S.K., Waye M.M., Mak T.C., Fong W.P., Fung K.P. (2006). Pheophorbide a, an active compound isolated from *Scutellaria barbata*, possesses photodynamic activities by inducing apoptosis in human hepatocellular carcinoma. Cancer Biol. Ther..

[34] Tang P.M., Liu X.Z., Zhang D.M., Fong W.P., Fung K.P. (2009). Pheophorbide a based photodynamic therapy induces apoptosis via mitochondrial-mediated pathway in human uterine carcinosarcoma. Cancer Biol. Ther..

[35] Tang P.M., Zhang D.M., Xuan N.H., Tsui S.K., Waye M.M., Kong S.K., Fong W.P., Fung K.P. (2009). Photodynamic therapy inhibits P-glycoprotein mediated multidrug resistance via JNK activation in human hepatocellular carcinoma using the photosensitizer pheophorbide a. Mol. Cancer.

[36] Bui-Xuan N.H., Tang P.M., Wong C.K., Fung K.P. (2010). Photo-activated pheophorbide-a, an active component of *Scutellaria barbata*, enhances apoptosis via the suppression of ERK-mediated autophagy in the estrogen receptor-negative human breast adenocarcinoma cells MDA-MB-231. J. Ethnopharmacol..

[37] Hoi S.W., Wong H.M., Chan J.Y., Yue G.G., Tse G.M., Law B.K., Fong W.P., Fung K.P. (2012). Photodynamic therapy of pheophorbide a inhibits the proliferation of human breast tumour via both caspase-dependent and -independent apoptotic pathways in in vitro and in vivo models. Phytother. Res..

[38] Ahn M.Y., Yoon H.E., Kwon S.M., Lee J., Min S.K., Kim Y.C., Ahn S.G., Yoon J.H. (2013). Synthesized pheophorbide a-mediated photodynamic therapy induced apoptosis and autophagy in human oral squamous carcinoma cells. J. Oral Pathol. Med..

[39] Ahn M.Y., Yoon H.E., Moon S.Y., Kim Y.C., Yoon J.H. (2017). Intratumoral photodynamic therapy with newly synthesized pheophorbide a in murine oral cancer. Oncol. Res..

[40] Cho M., Park G.M., Kim S.N., Amna T., Lee S., Shin W.S. (2014). Glioblastoma-specific anticancer activity of pheophorbide a from the edible red seaweed *Grateloupia elliptica*. J. Microbiol. Biotechnol..

[41] You H., Yoon H.E., Yoon J.H., Ko H., Kim Y.C. (2011). Synthesis of pheophorbide-a conjugates with anticancer drugs as potential cancer diagnostic and therapeutic agents. Bioorg. Med. Chem..

[42] Ruiz-Gonzalez R., Milan P., Bresoli-Obach R., Stockert J.C., Villanueva A., Canete M., Nonell S. (2017). Photodynamic synergistic effect of pheophorbide a and doxorubicin in combined treatment against tumoral cells. Cancers (Basel).

[43] Ohta S., Ono F., Shiomi Y., Nakao T., Aozasa O., Nagate T., Kitamura K., Yamaguchi S., Nishi M., Miyata H. (1998). Anti-herpes simplex virus substances produced by the marine alga, *Dunaliella primolecta*. J. Appl. Phycol..

[44] Ratnoglik S.L., Aoki C., Sudarmono P., Komoto M., Deng L., Shoji I., Fuchino H., Kawahara N., Hotta H. (2014). Antiviral activity of extracts from *Morinda citrifolia* leaves and chlorophyll catabolites, pheophorbide a and pyropheophorbide a, against hepatitis c virus. Microbiol. Immunol..

[45] Nicoletti M. (2016). Microalgae nutraceuticals. Foods.

[46] García J.L., de Vicente M., Galán B. (2017). Microalgae, old sustainable food and fashion nutraceuticals. Microbial Biotechnol..

[47] Lauritano C., Helland K., Riccio G., Andersen J.H., Ianora A., Hansen E.H. (2020). Lysophosphatidylcholines and chlorophyll-derived molecules from the diatom *Cylindrotheca closterium* with anti-inflammatory activity. Mar. Drugs.

[48] Yan X., Chuda Y., Suzuki M., Nagata T. (1999). Fucoxanthin as the major antioxidant in *Hijikia fusiformis*, a common edible seaweed. Biosci. Biotechnol. Biochem..

[49] Ye H., Zhou C., Sun Y., Zhang X., Liu J., Hu Q., Zeng X. (2009). Antioxidant activities in vitro of ethanol extract from brown seaweed *Sargassum pallidum*. Eur. Food Res. Technol..

[50] Sakata K., Yamamoto K.i., Ishikawa H., Yagi A., Etoh H., Ina K. (1990). Clhorophyllone-a, a new pheophorbide-a related compound isolated from *Ruditapes philippinarum* as an antioxidative compound. Tetrahedron Lett..

[51] Lanfer-Marquez U.M., Barros R.M.C., Sinnecker P. (2005). Antioxidant activity of chlorophylls and their derivatives. Food Res. Int..

[52] Cho M., Lee H.S., Kang I.J., Won M.H., You S. (2011). Antioxidant properties of extract and fractions from *Enteromorpha prolifera*, a type of green seaweed. Food Chem..

[53] Bui-Xuan N.H., Tang P.M., Wong C.K., Chan J.Y., Cheung K.K., Jiang J.L., Fung K.P. (2011). Pheophorbide a: A photosensitizer with immunostimulating activities on mouse macrophage raw 264.7 cells in the absence of irradiation. Cell. Immunol..

[54] Peloi L.S., Biondo C.E., Kimura E., Politi M.J., Lonardoni M.V., Aristides S.M., Dorea R.C., Hioka N., Silveira T.G. (2011). Photodynamic therapy for american cutaneous leishmaniasis: The efficacy of methylene blue in hamsters experimentally infected with *Leishmania (Leishmania) amazonensis*. Exp. Parasitol..

[55] Taylor V.M., Cedeno D.L., Munoz D.L., Jones M.A., Lash T.D., Young A.M., Constantino M.H., Esposito N., Velez I.D., Robledo S.M. (2011). In vitro and in vivo studies of the utility of dimethyl and diethyl carbaporphyrin ketals in treatment of cutaneous leishmaniasis. Antimicrob. Agents Chemother..

[56] Miranda N., Volpato H., da Silva Rodrigues J.H., Caetano W., Ueda-Nakamura T., de Oliveira Silva S., Nakamura C.V. (2017). The photodynamic action of pheophorbide a induces cell death through oxidative stress in *Leishmania amazonensis*. J. Photochem. Photobiol. B.

[57] Pangestuti R., Kim S.-K. (2011). Biological activities and health benefit effects of natural pigments derived from marine algae. J. Funct. Foods.

[58] Zhang Q., Libo L. (2018). Photodynamic combinational therapy in cancer treatment. JBUON.

[59] Carotenuto P., Pecoraro A., Palma G., Russo G., Russo A. (2019). Therapeutic approaches targeting nucleolus in cancer. Cells.

[60] Russo A., Saide A., Smaldone S., Faraonio R., Russo G. (2017). Role of ul3 in multidrug resistance in p53-mutated lung cancer cells. Int. J. Mol. Sci..

